# Is population frequency a useful criterion to assign pathogenicity to newly described mitochondrial DNA variants?

**DOI:** 10.1186/s13023-022-02428-0

**Published:** 2022-08-19

**Authors:** M. Pilar Bayona-Bafaluy, Ester López-Gallardo, Sonia Emperador, David Pacheu-Grau, Julio Montoya, Eduardo Ruiz-Pesini

**Affiliations:** 1 Departamento de Bioquímica, Biología Molecular y Celular, Universidad de Zaragoza, 50009 and 50013 Zaragoza, Spain; 2 Instituto de Investigación Sanitaria (IIS) de Aragón, 50009 Zaragoza, Spain; 3 Centro de Investigaciones Biomédicas en Red de Enfermedades Raras (CIBERER), 28029 Madrid, Spain; 4 Instituto de Biocomputación y Física de Sistemas Complejos (BIFI), Universidad de Zaragoza, 50018 Zaragoza, Spain

**Keywords:** Mitochondrial DNA, Pathological variants, Population frequency

## Abstract

Population frequency has been one of the most widely used criteria to help assign pathogenicity to newly described mitochondrial DNA variants. However, after sequencing this molecule in thousands of healthy individuals, it has been observed that a very large number of genetic variants have a very low population frequency, which has raised doubts about the utility of this criterion. By analyzing the genetic variation of mitochondrial DNA-encoded genes for oxidative phosphorylation subunits in 195,983 individuals from HelixMTdb that were not sequenced based on any medical phenotype, we show that rare variants are deleterious and, along with other criteria, population frequency is still a useful criterion to assign pathogenicity to newly described variants.

Population frequency is one of the most widely used criteria to help assigning pathogenicity to newly described mitochondrial DNA (mtDNA) variants. A genetic variant with a significant deleterious effect on the phenotype will hinder the survival or reproduction of the individual that carries it, will not be able to spread in the population and, therefore, its population frequency will be low. Despite the fact that the variants universally accepted to be pathological meet this criterion, a very important number of mtDNA genetic variants present in healthy individuals still have a very low population frequency. As an example, in a population of 195,983 individuals from HelixMTdb that were not sequenced based on any medical phenotype [1], we found that 67% out of 2903 different non-synonymous, homoplasmic variants were rare variants, with a population frequency ≤ 1/10,000 (0.01%). This fact raises doubts about the utility of this criterion to confirm the pathogenicity of newly described mtDNA variants [2].

To test whether rare, homoplasmic, non-synonymous mtDNA variants have a deleterious functional effect, we compared the ratio of non-synonymous and synonymous variants between groups of rare and non-rare variants from HelixMTdb (Fig. 1A). We found a statistically significant deficit of non-synonymous variants in the non-rare group. Synonymous variants have no effect on protein sequence and it was found that selection on these mtDNA positions is minimal [3]. On the contrary, our results suggested an important effect of selection on non-synonymous positions. They decreased in the non-rare group and accumulated in the rare group. The same analysis (non-synonymous/synonymous) on heteroplasmic variants did not show significant differences between rare and non-rare groups (Fig. 1A). This means that usually a rare, non-synonymous variant, at a low mutational load, will probably have not an important functional effect. On the other hand, homoplasmy is found in 88% out of 1081 non-rare, non-synonymous variants but only in 40% of 4876 rare, non-synonymous variants. Thus, if rare, non-synonymous variants have a deleterious effect will be more difficult for them to reach homoplasmy. A sampling bias might affect the frequency of these genetic variants. For example, HelixMTdb is overrepresented (91.2%) in Eurasian mtDNA N lineages [1], and mtDNA sequencing from other regions of the world, underrepresented until now in this sample, could change the frequency of some of the variants already described.

**Fig. 1:**
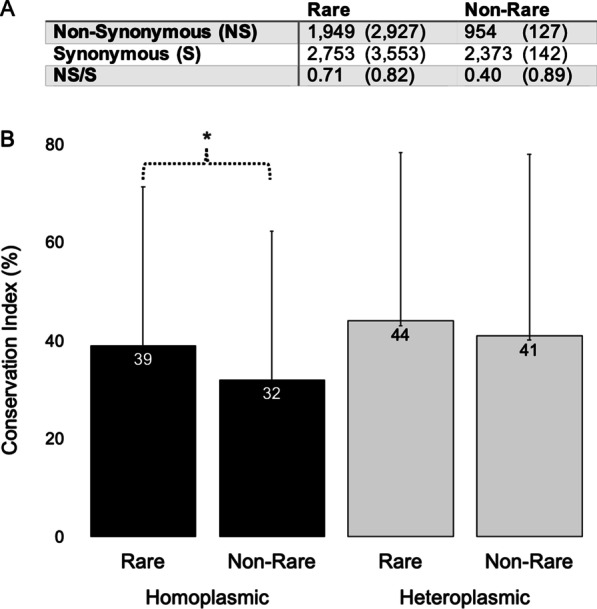
Analysis of mitochondrial DNA single nucleotide substitutions in protein genes. Rare, population frequency ≤ 1/10,000 (< 0.01%). **A** Absolute frequencies of mitochondrial DNA homoplasmic (heteroplasmic) variants. *P* < 0.00001 (*P* = 0.5321) (Fisher’s exact test). **B** Conservation index. Means ± Standard Deviations are shown. Asterisk, *P* = 1.2 E^− 07^ (Unpaired t-test)

The conservation index of a particular amino acid position in a protein is defined as the frequency of the reference amino acid at that particular protein position obtained from an alignment of reference orthologs from many species and is a commonly used attribute to determine pathogenicity of amino acid substitutions in mtDNA-encoded polypeptides [4]. A genetic variant with a significant deleterious effect on the phenotype will not be able to spread in the population and survive the speciation and, therefore, its interspecies frequency will be low. Therefore, the reference amino acid in that particular protein position will have a high conservation index.

We estimated the mean conservation index of amino acid positions (obtained from alignments of approximately 5000 reference orthologs from protists to mammals [4]) that presented homoplasmic mutations [36 ± 31.5 (2903)] and found that this conservation index was significantly lower (P = 4.9 E^− 18^, unpaired t-test) than that of heteroplasmic variants [44 ± 34.3 (3054)]. This result again suggested that variants in important amino acid positions were removed by natural selection. To confirm that rare, homoplasmic, non-synonymous variants have deleterious functional effects, we compared the rare and non-rare groups and found that conservation index of the rare group was significantly higher (Fig. 1B). Interestingly, there were not significant differences between rare and non-rare groups of heteroplasmic variants. Homoplasmic, non-synonymous variants with population frequencies < 0.01%, ≥ 0.01% but < 0.1%, or ≥ 0.1% affected protein positions with mean conservation indexes of 39, 33 and 28, respectively. The less frequent the variant, the higher the conservation index.

If we considered variants with a population frequency ≤ 1/100,000 (0.001%), still 23% (657) out of 2903 different non-synonymous, homoplasmic variants from the population of 195,983 individuals from HelixMTdb were very-rare variants. The results of comparisons of non-synonymous/synonymous ratios and the mean conservation indexes in homoplasmic variants of very-rare and non-very-rare groups were very similar to those previously shown for rare and non-rare variants (Fig. 2). However, these comparisons were also significant for heteroplasmic variants. Very-rare variants probably affect very important amino acid positions and, even in heteroplasmic state, they have a significant effect on the phenotype. All these results confirmed that very-rare or rare variants affected functionally important protein positions. Then, why are these variants found in healthy individuals?

**Fig. 2:**
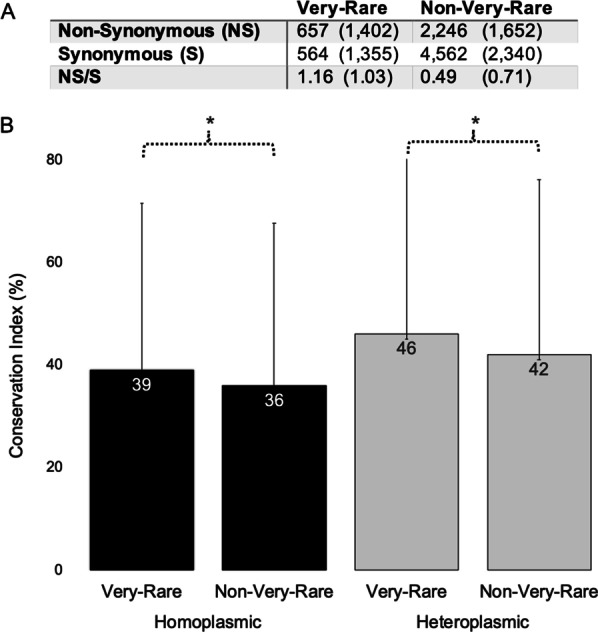
Analysis of mitochondrial DNA single nucleotide substitutions in protein genes. Very-rare, population frequency ≤ 1/100,000 (< 0.001%). **A** Absolute frequencies of mitochondrial DNA homoplasmic (heteroplasmic) variants. *P* < 0.00001 (*P* < 0.00001) (Fisher’s exact test). **B** Conservation index. Means ± Standard Deviations are shown. Asterisk, *P* ≤ 0.0335 (Unpaired t-test)

A first possibility is that many of these variants show incomplete penetrance. They are not enough to cause the pathological phenotype and require the effect of additional factors. For example, the m.3460G > A and m.11778G > A mutations in the *MT-ND1* and *MT-ND4* genes, that cause Leber hereditary optic neuropathy (LHON), have been found 14 and 78 times, respectively, in HelixMTdb. However, it was reported that mtDNA copy number differentiates LHON affected individuals harboring these mutations from the unaffected mutation carriers [5]. A second possibility is that, in individuals from HelixMTdb, the pathologic variant is compensated. Genetic compensation is probably relatively common for mtDNA variants [6]. As an example, the p.Val421Ala substitution in the mouse cytochrome c oxidase subunit 1 caused a significant reduction in this complex activity but a second p.Leu246Ile substitution in the same protein suppressed most of the defect [7]. The equivalent human variants (m.7165T > C and m.6639 C > A) are very rare, because they have not been reported in HelixMTdb. Moreover, conservation indexes of Leu246 and Val421 are 99 and 98%, respectively.

Our results suggest that population frequency is still a useful criterion to help assign pathogenicity to a newly described mtDNA variant. In any case, population frequency is not a definitive criterion to confirm pathogenicity of a new mtDNA non-synonymous variant and additional criteria are required, such as conservation index and others [2]. In our opinion, a functional evaluation in homogenous environmental and genetic conditions, such as single fiber analyses or improved cybrids studies, is key for the pathogenicity assignment [8]. However, functional explorations require much more time and work than DNA sequencing and many variants candidate to be the ethiologic factor of the disease remain incompletely characterized, being a potential cause of future problems [9]. Even with the current detailed criteria, the process of variant classification is still more art than science [9]. As it has been suggested, the difficulties in the evaluation of genetic variants call for the birth of a new discipline, the interpretive medical genomics, that requires expertise in genetics, bioinformatics, statistics, and in vitro and in vivo functional analysis [10].

## Data Availability

The datasets used and analysed during the current study are available from the corresponding author on reasonable request.
